# Self-reported exercise and longitudinal outcomes in cystic fibrosis: a retrospective cohort study

**DOI:** 10.1186/1471-2466-14-159

**Published:** 2014-10-06

**Authors:** Joseph M Collaco, Scott M Blackman, Karen S Raraigh, Christopher B Morrow, Garry R Cutting, Shruti M Paranjape

**Affiliations:** Eudowood Division of Pediatric Respiratory Sciences, Johns Hopkins Medical Institutions, David M. Rubenstein Building, 200 North Wolfe Street, 21287 Baltimore, MD USA

**Keywords:** Cystic fibrosis, Lung function, FEV_1_, Body mass index, Exercise

## Abstract

**Background:**

Cystic fibrosis (CF) is characterized by recurrent respiratory infections and progressive lung disease. Whereas exercise may contribute to preserving lung function, its benefit is difficult to ascertain given the selection bias of healthier patients being more predisposed to exercise. Our objective was to examine the role of self-reported exercise with longitudinal lung function and body mass index (BMI) measures in CF.

**Methods:**

A total of 1038 subjects with CF were recruited through the U.S. CF Twin-Sibling Study. Questionnaires were used to determine exercise habits. Questionnaires, chart review, and U.S. CF Foundation Patient Registry data were used to track outcomes.

**Results:**

Within the study sample 75% of subjects self-reported regular exercise. Exercise was associated with an older age of diagnosis (*p* = 0.002), older age at the time of ascertainment (*p* < 0.001), and higher baseline FEV_1_ (*p* = 0.001), but not *CFTR* genotype (*p* = 0.64) or exocrine pancreatic function (*p* = 0.19). In adjusted mixed models, exercise was associated with both a reduced decline in FEV_1_ (*p* < 0.001) and BMI Z-score (*p* = 0.001) for adults, but not children aged 10–17 years old.

**Conclusions:**

In our retrospective study, self-reported exercise was associated with improved longitudinal nutritional and pulmonary outcomes in cystic fibrosis for adults. Although prospective studies are needed to confirm these associations, programs to promote regular exercise among individuals with cystic fibrosis would be beneficial.

**Electronic supplementary material:**

The online version of this article (doi:10.1186/1471-2466-14-159) contains supplementary material, which is available to authorized users.

## Background

Cystic fibrosis (CF) is a life-limiting single gene disorder caused by mutations in the cystic fibrosis transmembrane conductance regulator gene (*CFTR*) leading to progressive lung function decline. As half of the variation in CF lung disease is secondary to non-genetic factors [1, 2], examining the role of environmental factors is critical in reducing the morbidity and mortality associated with CF. Although a number of factors have been shown to have an impact on lung function in CF, including secondhand smoke exposure [3–5], health insurance [6–9], household income [10], adherence [11], air pollution [12], and ambient temperature [13], only two of these are readily modifiable: secondhand smoke and adherence. Another potential modifiable factor to improve outcomes may be exercise.

There have been a number of well designed randomized controlled trials of exercise among individuals with CF to determine the effects of exercise on lung function and nutritional status. These trials, lasting from 1 week to 3 years, have employed aerobic exercise, resistance training, or a combination of both. Some studies have demonstrated benefits to FEV_1_ [14, 15], and some have seen promising trends [16, 17], while others have not seen any benefit [18–20]. Similarly, some studies have seen a benefit to FVC [15–18], while others have not [14, 19, 20]. The mixed results of these studies may be related to the differing ages of participants or the differing types and duration of exercise regimens utilized, although it should be noted that no difference in FEV_1_ was seen in a 1 year trial of resistance training vs. aerobic training in CF. [21] Another reason for the differing results may be statistical power, as these studies range from 13 to 65 participants. A hypothetical study with 30 cases and 30 controls with an assumed baseline FEV_1_ percent predicted rate of decline of 2.0 ± 1.0% per year would only have a statistical power of 62% to detect a difference of 0.5% decline per year. However, a recent larger prospective study of exercise in children with cystic fibrosis (n = 212) did see an association between increased habitual physical activity and a reduced decline in FEV_1_ [22].

Many individuals with CF also have exocrine pancreatic insufficiency leading to lifelong malabsorption and poor weight gain. Thus a potential concern is the increased caloric expenditure resulting from exercise. Limited data suggest that individuals with CF expend more energy with exercise than do healthy controls independent of baseline resting energy expenditures [23]. The results are mixed for the effects of exercise on nutritional status in randomized controlled trials; at least one study has seen benefits for body mass index (BMI) or ideal weight-for-height for certain types of exercise [14], one study observed a trend towards benefit [16], and other studies have not seen any benefit [18–20].

In the absence of a larger randomized controlled trial, we examined longitudinal respiratory and nutritional outcomes in a large, well characterized sample of individuals with cystic fibrosis. Exercise was self-reported representing a real-world scenario of asking patients whether they participate in regular exercise. Using data from the CF Twin-Sibling Study, we hypothesized that exercise would be associated with a reduced decline in lung function (FEV_1_) and improved nutritional status (body mass index Z-scores) over time for individuals who exercised compared to those who did not. Secondary analyses included assessing the prevalence of wheezing in individuals who exercised versus those who did not as we hypothesized that individuals reporting wheezing with exercise would be less likely to participate in exercise. Given our study sample size, we were also able to examine whether specific subgroups of subjects were more likely to benefit from exercise (i.e., males vs. females, children vs. adults, etc.).

## Methods

### Study sample

All subjects were part of the CF Twin-Sibling Study (n = 1038 individuals in 575 families) and were recruited from CF centers in the United States (n = 952), Australia (n = 48), Israel (n = 24), and United Kingdom (Scotland)(n = 14) between 2000 and 2013 on the basis of having CF and having a twin or sibling with CF. The CF Twin-Sibling Study was designed to assess genetic and environmental modifiers of clinical outcomes in CF. Written consent was obtained from all subjects and the study was approved by the Johns Hopkins University Institutional Review Board (Protocol #NA_00035659). Baseline questionnaires were obtained upon enrollment and included (cross-sectional) questions on whether exercise was routinely undertaken. English-language questionnaires included questions (Additional file 1: Table S1) on whether various types of exercise were undertaken (i.e., competitive, recreational, or both), which was tabulated for subjects ≥10 years of age. The questionnaires used are not validated and not age-specific; caregivers routinely completed questionnaires for children less than 18 years of age.

### Demographics

Age for each subject was defined by the date the questionnaire was completed. Race/ethnicity was self-defined with subjects reporting any non-white ancestry defined as non-white. Pancreatic sufficiency was defined by genotype data (n = 911) as having one or more “pancreatic sufficient” mutations, and by clinical data (n = 106) where genotype data was indeterminate or not available. We were unable to ascertain pancreatic function status for 21 subjects.

### Outcome ascertainment

The presence or absence of wheezing with exertion was defined by self-report. Pulmonary function test and body mass index outcome data was collected through chart review with data supplementation from the U.S. Cystic Fibrosis Foundation Patient Registry. Longitudinal data from non-U.S. locations were not necessarily available. All raw FEV_1_ (liters) measurements were converted into FEV_1_ percent predicted values as defined by the U.S. CF Foundation [24, 25]. Baseline FEV_1_ for a subject was defined as the average FEV_1_ of all measurements obtained the 12 months preceding or after the exercise questionnaire date. Change in lung function over time was calculated from linear regression of all FEV_1_ percent predicted measurements in the 5 years following questionnaire completion for subjects with a minimum of 4 FEV_1_ values taken over a period of no less than 2 years. Body mass indices were converted into Z-scores using CDC reference equations [26]. Baseline BMI Z-score and change in BMI Z-score were calculated in a similar manner to FEV_1_.

### Statistical analysis

**Figure 1:**
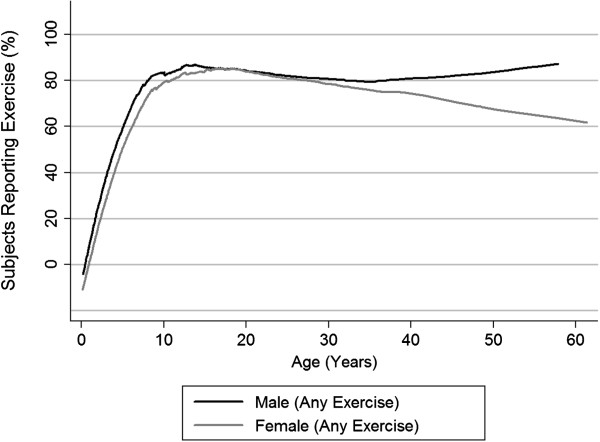
**Exercise participation vs. age.** Lowess regression of percentage of subjects reporting specific types of exercise versus age at the time of ascertainment.

Differences in baseline characteristics and clinical outcomes were compared using *T*-tests for continuous variables and Pearson’s chi-square for categorical variables. To assess the effect of self-reported exercise on longitudinal changes in lung function while accounting for confounders, we employed mixed-effects regression modeling for longitudinal repeated measures with unstructured covariance and random effects for intercept and slope. For longitudinal effects, we only assessed subjects over the age of 10 years based on when rates of exercise participation stabilized (Figure 1). Baseline FEV_1_, sex, age of diagnosis, and age of questionnaire completion were included as potential confounders as these variables differed between those who reported exercising and those who did not (Table 1). All models used a dependent variable of FEV_1_ percent predicted consisting of all FEV_1_ measurements obtained over 5 years following the time of ascertainment of exercise status (t = 0). The independent variables were time since ascertainment, exercise status, and a time-exercise interaction term. Exercise status was defined as no exercise = 0 and any exercise = 1 for the any exercise models and as no competitive exercise = 0 and any competitive exercise = 1 for the competitive exercise models. All adjusted models included the covariates of baseline FEV_1_ percent predicted age at diagnosis, age at t = 0, and sex. We tested whether different subgroups of the study sample (females, adults, and subjects with reduced lung function) had different changes in lung function with exercise using time-exercise-sex, time-exercise-adult (adults being ≥18 years old), and time-exercise-reduced lung function (reduced lung function being baseline FEV_1_ < 80%) interaction terms. The interaction for sex (female = 1; male = 0) model included a time-exercise-sex interaction term. The interaction for age (≥18yo = 1; <18yo = 0) model included both the term dichotomous term for age and a time-exercise-dichotomous age interaction term. The interaction for baseline FEV_1_ (≥80% = 0; <80% = 1) model included both the term dichotomous term for baseline FEV_1_ and a time-exercise-dichotomous FEV_1_ interaction term. *P* values of less than 0.05 were considered statistically significant. Similar analyses were performed for BMI Z-scores, except both baseline BMI Z-score and baseline FEV_1_ were retained as covariates. In addition, the interaction term for time-exercise-reduced BMI Z-score was defined by reduced BMI-Z score < 0. All analyses were performed using Stata IC 11.0 (StataCorp LP, College Station, TX).

**Table 1:** **Study sample demographics and clinical outcomes**

Mean ± S.D. [range]	Entire study sample	No exercise	Any exercise	***P***value
**n**	1038	264	774	-
**Sex** (% male)	51.8	47.0	53.5	0.07
**Race/Ethnicity** (% non-white)	9.2	11.0	8.5	0.23
	(n = 1036)	(n = 263)	(n = 773)	
**Age at Diagnosis** (years)	2.5 ± 5.7	1.5 ± 4.7	2.8 ± 6.0	0.002
	[0, 52]	[0, 51]	[0, 52]	
	(n = 1025)	(n = 259)	(n = 766)	
***CFTR*** **Genotype** (% F508del homozygotes)	45.2	46.4	44.7	0.64
	(n = 1023)	(n = 263)	(n = 760)	
**Exocrine Pancreatic Function** (% insufficient)	83.3	85.9	82.4	0.19
	(n = 1017)	(n =256)	(n = 761)	
**Age at Ascertainment of Exercise Status** (years)	13.8 ± 10.2	10.1 ± 11.1	15.0 ± 9.6	<0.001
	[0.1, 61.3]	[0.1, 61.3]	[1.4, 57.8]	
**Wheezing Reported with Exercise** (% yes)	23.4	22.0	23.9	0.54
	(n = 995)	(n = 250)	(n = 745)	
**Baseline FEV**_**1**_ **% Predicted** ^**†**^	84.5 ± 22.5	78.0 ± 26.7	85.6 ± 21.5	0.001
	[19.7, 145.8]	[19.7, 145.8]	[21.4, 137.6]	
	(n = 766)	(n = 115)	(n = 651)	
**Change in FEV**_**1**_ **% Predicted/Year** ^**††**^	-1.33 ± 4.65	-0.99 ± 5.48	-1.39 ± 4.47	0.44
	[-26.4, 24.3]	[-26.4, 23.4]	[-23.2, 24.3]	
	(n = 558)	(n = 94)	(n = 464)	
**Baseline BMI Z-score** ^**†**^	-0.15 ± 0.99	-0.12 ± 1.05	-0.16 ± 0.98	0.55
	[-4.72, 3.12]	[-4.06, 3.12]	[-4.72, 2.50]	
	(n = 904)	(n = 207)	(n = 697)	
**Change in BMI Z-score/Year** ^**††**^	-0.01 ± 0.20	0.02 ± 0.23	-0.01 ± 0.19	0.16
	[-1.02, 0.83]	[-1.02, 0.70]	[-0.82, 0.83]	
	(n = 591)	(n = 134)	(n = 457)	

^**†**^All FEV_1_ % predicted values were generated using U.S. CFF guidelines. All BMI Z-scores were generated using CDC percentiles. Baseline function represents the mean of all measurements obtained within 1 year before and after the age of exercise status ascertainment.

^**††**^Change in function represents the predicted change using linear regression and all measurements obtained 5 years after the age of exercise ascertainment for each subject. Only subjects with a minimum of 4 measurements and a minimum of 2 years of data were included.

## Results

### Demographics

Of the 1038 subjects in the study sample, 774 (74.6%) reported performing exercise and (25.4%) reported no exercise (Table 1). For the 605 subjects over the age of 10 years at the time of exercise ascertainment, 278 (46.0%) reported participating in any competitive exercise, 235 (38.8%) reported participating in recreational exercise only, and 92 (15.2%) reported no exercise. As seen in Figure 1, participation rates in any exercise increased steadily until 10 years of age, then reached a plateau of ~80%, which remained relatively constant in males, but gradually decreased in females. There were no differences in the percentage of non-white individuals (*p* = 0.23) by exercise participation. There was a slight trend to more males (53%) reporting exercise than females (47%; *p* = 0.07). The group that reported no exercise had a younger age of diagnosis (1.5 ± 4.7 years) than the exercise group (2.8 ± 6.0 years; *p* = 0.002); however, no differences were seen with exocrine pancreatic function status (*p* = 0.19) or *CFTR* genotype (frequency of *F508del* homozygotes)(*p* = 0.64). The group that reported no exercise had a younger age of exercise ascertainment (10.1 ± 11.1 years) than the exercise group (15.0 ± 9.6 years; *p* < 0.001). Between countries, there were differences in the frequency of *F508del* homozygotes (U.S., 45.9%; Australia, 62.2%; Israel, 0%; U.K., 21.4%; *p* < 0.001), frequency of exocrine pancreatic function (U.S., 84.2%; Australia, 81.3%; Israel, 66.7%; U.K., 57.1%; *p* = 0.006), and age at the time of exercise ascertainment (U.S., 14.1 years; Australia, 8.4; Israel, 16.2; U.K., 8.3; *p* < 0.001), but no other demographic differences (Additional file 1: Table S2).

### Respiratory outcomes

There was no difference in the proportion of subjects who reported wheezing with exertion between those who did not routinely exercise (22.0%) vs. those did (23.9%)(Table 1). Those who reported exercise had a higher baseline FEV_1_ percent predicted (85.6 ± 21.5%) than those who did not (78.0 ± 26.7%; *p* = 0.001). Similar findings were seen in *F508del* homozygotes where those who reported any exercise had a higher mean baseline FEV_1_ (84.5 ± 21.7; n = 283) compared to the no exercise group (73.7 ± 27.0; n = 53; *p* = 0.002). For the entire study sample, there were no differences in the rates of lung function decline in the 5 years following ascertainment of exercise status between those who exercised vs. those who did not.

**Figure 2:**
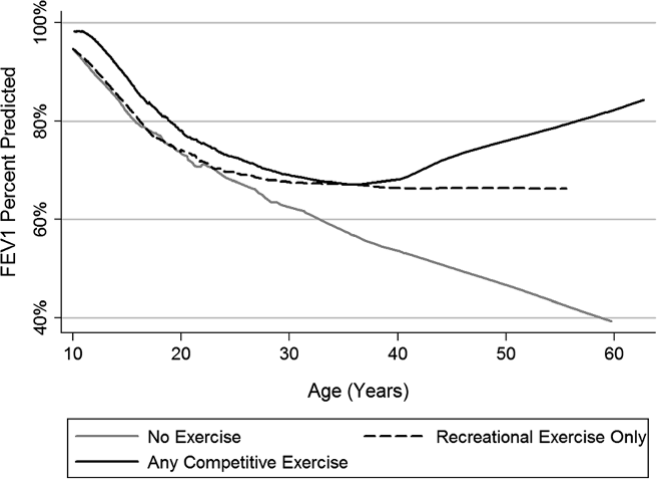
**Lung function and exercise.** Average FEV_1_ Percent Predicted Trajectories by Exercise Group. Lowess regression of lung function constructed using 2 datapoints per subject (≥10 years old at the time of exercise ascertainment), specifically the baseline FEV_1_ and FEV_1_ at the end of follow-up based on lung function decline. Subjects were limited to these two points each to avoid overweighting subjects with substantially more FEV_1_ measurements. Estimates after 40 years of age may not be as robust owing to fewer older subjects. Although those reporting competitive exercise always appear to have higher lung function than those reporting no exercise, those report recreational exercise only appear to be similar to the no exercise group between ages 10 and 20 years old, then resemble the competitive exercise group in adulthood.

To account for differences in baseline FEV_1_, differences in age at the time of exercise status ascertainment, and sex-related and age-at-diagnoses differences in exercise participation, we employed mixed model regressions including these factors as covariates. We also limited all mixed model samples to subjects ≥10 years of age at exercise ascertainment as exercise participation rates appeared to plateau after 10 years of age (Figure 1). After these adjustments, we found that the baseline decline in FEV_1_ percent predicted per year for 389 subjects where complete data were available was -2.03% per year (Table 2). An increase of 0.35% per year with exercise compared to the adjusted baseline FEV_1_ decline was observed, giving a reduced FEV_1_ decline of -1.68% per year with any exercise, but this was not statistically significant (*p* = 0.59). We also tested several interaction terms to assess whether differences in FEV_1_ decline with exercise were observed by sex, age, or severity of lung disease, more specifically to determine if certain subgroups would be more likely to benefit from exercise. We found that exercising adults with CF (≥18 years old) would experience a smaller decline in FEV_1_ (-0.50% /year) compared to exercising children (10–17 years old)(-2.48% /year)(*p* < 0.001), suggesting that adults may receive more of a benefit than children from exercise (Additional file 1: Figure S1). A graphic depiction of average lung function decline is shown in Figure 2 for each of the three exercise groups, showing that those reporting exercise do have higher lung function at earlier ages with a decreased decline in adulthood. An interaction term for sex was not significant. We did observe that exercising subjects with a reduced baseline FEV_1_ (<80%) experienced a smaller decline in FEV_1_ (-1.05%/year) compared to exercising subjects with a baseline FEV_1_ ≥ 80% (-2.13%/year)(*p* < 0.001). We also performed the same analysis comparing those who reported any competitive exercise vs. recreational only or no exercise to determine whether possibly more intense exercise might result in greater benefits (Table 2). No statistical reduction in the rate of FEV_1_ decline was observed, except as before, competitively exercising adults with CF (≥18 years old) would experience less of decline in FEV_1_ compared to competitively exercising children (10–17 years old)(*p* = 0.001).

**Table 2:** **Change in lung function with exercise with interaction term analyses (Subjects ≥ 10 years old)**

Models: n = 389 subjects		Estimated FEV_1_% at time = 0 without exercise*	Estimated FEV_1_% at time = 0 with exercise* (***p***value)	Baseline Change in FEV_1_% per year (***p***value)	Change in FEV_1_% over baseline per year with exercise (***p***value)	Change in FEV_1_% over baseline per year for interaction (***p***value)
**Any Exercise**	**Unadjusted Model (No Covariates)**	74.8	82.6 (0.020)	-2.01 (0.001)	0.28 (0.67)	-
	**Adjusted Model**	81.7	81.6 (0.95)	-2.03 (0.001)	0.35 (0.59)	-
	**Adjusted Model + Interaction for Sex**	81.9	81.8 (0.92)	-2.03 (0.001)	0.05 (0.94)	0.67 (0.17)
	**Adjusted Model + Interaction for Age**	82.2	82.1 (0.93)	-2.03 (0.001)	-0.45 (0.50)	1.98 (<0.001)
	**Adjusted Model + Interaction for Baseline FEV**_**1**_	81.6	81.4 (0.87)	-2.03 (0.001)	-0.10 (0.88)	1.08 (0.028)
**Competitive Exercise**	**Unadjusted Model (No Covariates)**	77.9	85.7 (0.001)	-1.70 (<0.001)	-0.16 (0.74)	-
	**Adjusted Model**	81.7	81.6 (0.98)	-1.64 (<0.001)	-0.18 (0.70)	-
	**Adjusted Model + Interaction for Sex**	81.7	81.7 (0.98)	-1.64 (<0.001)	-0.28 (0.61)	0.22 (0.73)
	**Adjusted Model + Interaction for Age**	81.1	81.1 (0.93)	-1.64 (<0.001)	-0.84 (0.11)	1.83 (0.004)
	**Adjusted Model + Interaction for Baseline FEV**_**1**_	81.7	81.6 (0.93)	-1.65 (<0.001)	-0.49 (0.35)	0.87 (0.17)

*Estimated intercepts were calculated using the regression coefficients assuming the subject was male and had the mean values for baseline FEV_1_ percent predicted (80.8 ± 22.2), age of diagnosis (3.6 ± 7.5 years), and age at time of exercise ascertainment (19.5 ± 9.4 years)(n = 389). The estimated FEV_1_ values are not statistically different for the adjusted models owing to the adjustment for baseline FEV_1_.

### Nutritional outcomes

There were no differences in baseline BMI Z-score or BMI Z-score change over time between those who reported exercise, those who reported recreational only exercise, and those who reported competitive exercise. Again to account for potential confounders, we employed mixed model regressions including the baseline BMI Z-score, baseline FEV_1_, sex, age at diagnosis, and age at the time of exercise status ascertainment as covariates. After these adjustments, we found that the change in BMI Z-score per year for 366 subjects ≥10 years of age where complete data were available was not statistically different from zero (*p* = 0.08; Table 3). In addition, no statistically significant increase or decrease in BMI Z-score was seen with any exercise (*p* = 0.30). We also tested several interaction terms to determine whether differences in BMI Z-scores over time with exercise were observed by sex, age, or severity of lung disease. We found that exercising adults with CF (≥18 years old) would experience 0.08 Z-score per year increase compared to exercising children (10–17 years old)(*p* = 0.001), again suggesting that adults may receive more of a benefit than children from exercise (Additional file 1: Figure S2). Interaction terms for sex and baseline BMI Z-score were not significant. As before we performed the same analysis comparing those who reported any competitive exercise vs. recreational only or no exercise (Table 3). In this case we found that for competitively exercising subjects with a BMI less than the 50th percentile (BMI Z-score < 0) would experience 0.06 Z-score per year increase compared to competitively exercising subjects with a BMI greater than the 50th percentile (*p* = 0.044). Interaction terms for sex and adulthood were not significant.

**Table 3:** **Change in BMI Z-scores with exercise with interaction term analyses (subjects ≥ 10 years old)**

Models: n = 366 subjects		Estimated BMI Z-score at time = 0 without exercise*	Estimated BMI Z-score at time = 0 with exercise*(***p***value)	Baseline decline in BMI Z-score per year (***p***value)	Change in BMI Z-score over baseline per year with exercise (***p***value)	Change in BMI Z-score over baseline per year for interaction (***p***value)
**Any Exercise**	**Unadjusted Model (No Covariates)**	-0.36	-0.30 (0.65)	-0.05 (0.08)	0.03 (0.29)	-
	**Adjusted Model**	-0.30	-0.30 (0.96)	-0.05 (0.08)	0.03 (0.30)	-
	**Adjusted Model + Interaction for Sex**	-0.29	-0.30 (0.93)	-0.05 (0.08)	0.02 (0.48)	0.02 (0.36)
	**Adjusted Model + Interaction for Age**	-0.32	-0.32 (0.91)	-0.05 (0.08)	0.001 (0.98)	0.08 (0.001)
	**Adjusted Model + Interaction for Baseline BMI Z-Score**	-0.24	-0.25 (0.92)	-0.05 (0.08)	0.03 (0.92)	0.04 (0.07)
**Competitive Exercise**	**Unadjusted Model (No Covariates)**	-0.41	-0.18 (0.021)	-0.03 (0.025)	0.02 (0.27)	-
	**Adjusted Model**	-0.30	-0.31 (0.72)	-0.03 (0.024)	0.03 (0.25)	-
	**Adjusted Model + Interaction for Sex**	-0.30	-0.31 (0.72)	-0.03 (0.024)	0.02 (0.38)	0.01 (0.80)
	**Adjusted Model + Interaction for Age**	-0.30	-0.31 (0.69)	-0.03 (0.024)	0.005 (0.85)	0.06 (0.06)
	**Adjusted Model + Interaction for Baseline BMI Z-Score**	-0.24	-0.26 (0.67)	-0.03 (0.023)	-0.01 (0.68)	0.06 (0.044)

*Estimated intercepts were calculated using the regression coefficients assuming the subject was male and had the mean values for baseline BMI Z-score (-0.30 ± 0.95), baseline FEV_1_ percent predicted (80.3 ± 22.0), age of diagnosis (3.5 ± 7.2 years), and age at time of exercise ascertainment (19.4 ± 9.3 years)(n = 366). The estimated BMI Z-score values are not statistically different for the adjusted models owing to the adjustment for baseline BMI Z-scores.

## Discussion

In our questionnaire-based study we found that approximately 75% of individuals with cystic fibrosis reported exercising on a regular basis. Exercise rates climbed through childhood and appeared to plateau in early adolescence, suggesting that exercise patterns may become fixed during childhood for cystic fibrosis. Exercise was associated with a higher baseline lung function, but not BMI Z-score. Using mixed models with interaction terms adjusted for baseline lung function, we found that adults who reported exercising had a less rapid decline in lung function and BMI Z-scores compared to their non-exercising peers. We also observed that subjects with reduced FEV_1_ who perform any exercise may benefit disproportionately better than those with better lung function in terms of FEV_1_ decline. Likewise, we observed that subjects with reduced BMI Z-scores who perform competitive exercise may benefit disproportionately better than those with better BMI Z-scores in terms of BMI Z-score changes over time. We did not see any differences in self-reported wheezing with exertion suggesting that wheezing with exertion may not be limiting regular exercise participation or that it may be well-controlled with medical therapy.

Although many previous randomized control trials of aerobic exercise have not seen a significant benefit to FEV_1_ with exercise, we did observe a reduced decline in FEV_1_ for adults who exercised compared to those who did not. This may be due to the fact that short-term randomized controlled trials may be capturing the physiological response to training, whereas our study is attempting to capture the long-term effects of more continuous exercise. Similar to our study, the longest randomized control trial (3 years) did see a trend towards a decreased rate of FEV_1_ decline in those assigned to the aerobic exercise group (-1.46% per year) vs. those in the control group (-3.47% per year; *p* = 0.07) [17]. Also, the effects of exercise may be accumulated over many years, which may be why many of the randomized controlled trials which predominantly enrolled participants <18yo do not see a benefit with FEV_1_ or nutritional measures, and these benefits are only observed in adults in our study. An alternative hypothesis is that children may perform a different mix of aerobic vs. anaerobic activities than adults, which may have different consequences. Finally, it should be noted that Schneiderman *et al.* did see and association between exercise and a reduced decline in lung function among children with cystic fibrosis, which may reflect a different means of exercise ascertainment (HAES questionnaire) or differences in care within the study locations in Canada [22].

We did observe that females were less likely to report performing exercise than their male counterparts starting at 25–30 years old. Previously published cross-sectional studies suggest that differences in activity levels between sexes may occur as early as pubescence [27]. This finding may contribute to sex-specific differences in survival observed in CF. [28, 29] Although at least one study has reported that rates of FEV_1_ decline were associated with activity levels in girls, but not in boys [29], we did not see any sex-specific benefits of exercise in interaction modeling and the response to exercise may be more a function of fitness rather than gender [30]. We also did not observe any differences in exercise rates by *CFTR* genotype; however, *CFTR* genotype may play a role in fitness level independent of lung function [31].

Strengths of this study include a well characterized population of individuals with CF with corresponding longitudinal phenotype data. Assessing exercise participation through a yes/no question does mimic real-world clinical practice where time may limit the use of validated questionnaires for exercise participation. However, assessing exercise in this categorical manner is the primary limitation of our study and may result in an overestimate of exercise rates. In our study sample, self-reported exercise rates reached 80% in adolescence and early adulthood. This compares to the 2010 CDC on physical activity where only 64.5% of U.S. adults were physically active as defined by ≥150 minutes/week of moderate-intensity activity or ≥75 minutes/week of vigorous activity [32]. It is possible that self-reported rates of exercise in cystic fibrosis may also be higher if exercise is being used as a form of airway clearance as part of the regular maintenance treatment regimen, but may also represent a form of social desirability bias when answering questionnaires. For these reasons we also assessed the effects of competitive activity, which may be a less biased measure in our population as 46% of our CF subjects over the age of 10 years reported any competitive activity, which compares to 43.5% of the general population reporting being highly active, which corresponds to ≥300 minutes/week of moderate-intensity activity or ≥150 minutes/week of vigorous activity [32]. Nevertheless, the potential misclassification bias associated self-report of exercise may lead to being underpowered to detect the effects of exercise. We also had the opportunity to assess exercise at one point in time and our data suggests that exercise patterns may change over time, at a minimum in young children as rates of exercise increase during childhood. Our data also does not include any quantitative assessment of exercise intensity or exercise types (e.g., aerobic vs. anaerobic), which could also affect outcomes. Lastly, although we did adjust for baseline lung function within our regression models, it is still possible that we overestimated the benefits of exercise as there may be unmeasured confounders that are associated with higher baseline lung function that reduce the rate of decline.

## Conclusions

In conclusion, we found that adults may have more of a benefit from regular exercise than children aged 10–17 years old, both in terms of slowing lung function decline and preserving body mass index in CF. However, as exercise patterns may be established prior to young adulthood for individuals with CF, there may be some benefit for pediatric clinicians to promote regular exercise habits, despite the many barriers to adherence to exercise [33]. We also did not see any decline or reduced BMI Z-scores with exercise, suggesting that individuals with CF who exercise are likely able to increase their caloric intake to meet the increased expenditures with exercise. Previous studies have found that individuals with the lowest fitness levels may see the most benefits with exercise [30, 34], and we did observe disproportionate benefits for those with reduced FEV_1_ and/or reduced BMI Z-scores. While there is a theoretical need for large prospective studies to confirm these findings for individuals with CF, we believe the longitudinal outcome benefits associated with exercise outweigh the risks.

## Electronic supplementary material

Additional file 1: Figure S1: Rate of FEV_1_ Decline in Children and Adults with and without Exercise. **Figure S2:** Rate of BMI Z-Score Decline in Children and Adults with and without Exercise. **Table S1:** Relevant Exercise Questions from CF Twin and Sibling Study Personnel Questionnaire Form. **Table S2:** Study Sample Demographics by Location. (DOC 112 KB)

## Abbreviations

BMI: Body mass index
CF: Cystic fibrosis
FEV1: Forced expiratory volume in 1 second.
