# Supplementary material for: Self-reported exercise and longitudinal outcomes in cystic fibrosis: a retrospective cohort study
Source: BMC Pulm Med. 2014 Oct 6;14:159. doi: 10.1186/1471-2466-14-159 (PMC4195986; doi:10.1186/1471-2466-14-159)
Supplement: Supplementary file 1 — Additional file 1: Figure S1: Rate of FEV1 Decline in Children and Adults with and without Exercise. Figure S2: Rate of BMI Z-Score Decline in Children and Adults with and without Exercise. Table S1: Relevant Exercise Questions from CF Twin and Sibling Study Personnel Questionnaire Form. Table S2: Study Sample Demographics by Location. (DOC 112 KB) [file 12890_2014_594_MOESM1_ESM.doc]

**Additional file1: Figure S1:** Rate of FEV_1_ Decline in Children and Adults with and without Exercise. Lines depicted are based on the adjusted regression model with the age interaction term in Table 3 using hypothetical males with mean baseline FEV_1_ values and age of diagnosis for the regression model populations. Age at exercise ascertainment was arbitrarily set at 15 years old for a child and 25 years old for an adult for this figure.


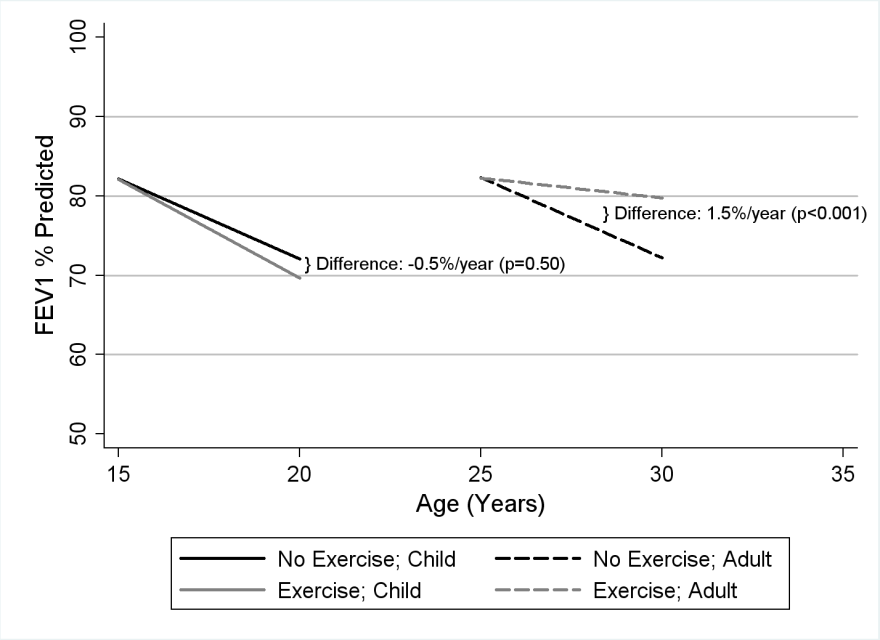


**Additional file1: Figure S2:** Rate of BMI Z-Score Decline in Children and Adults with and without Exercise. Lines depicted are based on the adjusted regression model with the age interaction term in Table 5 using hypothetical males with mean baseline BMI Z-score values, FEV_1_ values, and age of diagnosis for the regression model populations. Age at exercise ascertainment was arbitrarily set at 15 years old for a child and 25 years old for an adult for this figure.


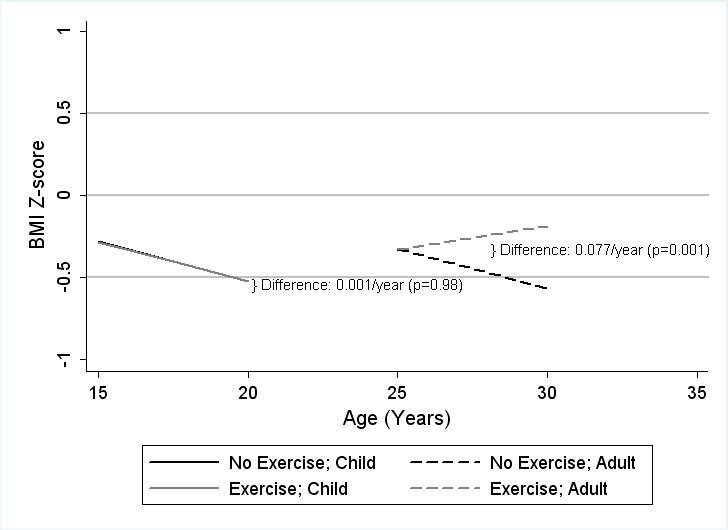


**Additional file1: Table S1:** Relevant Exercise Questions from CF Twin and Sibling Study Personnel Questionnaire Form

| **Physical Activity** | **(please circle)^1^** |
| --- | --- |
| Competitive sports | Y / N / U |
| Recreational sports/Physical Fitness | Y / N / U |

| **Miscellaneous Medical History Information** | **( please circle)^1^** |
| --- | --- |
| Wheeze with exercise | Y / N / U |

^1^Y = Yes, N = No, U = Unknown

**Additional file1: Table S2:** Study Sample Demographics by Location

| Mean ± S.D.  [Range] | **United States** | **Australia** | **Israel** | **United Kingdom (Scotland)** | ***P* value** |
| --- | --- | --- | --- | --- | --- |
| **n** | 952 | 48 | 24 | 14 | - |
| **Sex** (% male) | 51.8 | 47.9 | 45.8 | 78.6 | 0.19 |
| **Race/Ethnicity** (% non-white) | 9.8  (n = 950) | 4.2 | 0.0 | 0.0 | 0.13 |
| **Age at Diagnosis** (years) | 2.5 ± 5.8  [0, 52]  (n = 942) | 1.5 ± 3.8  [0, 16]  (n = 47) | 4.8 ± 6.4  [0, 17.1]  (n = 22) | 1.7 ± 3.1  [0, 11] | 0.15 |
| ***CFTR* Genotype** (% F508del homozygotes) | 45.9  (n = 940) | 62.2  (n = 45) | 0.0 | 21.4 | <0.001 |
| **Exocrine Pancreatic Function** (% insufficient) | 84.2  (n = 931) | 81.3 | 66.7 | 57.1 | 0.006 |
| **Age at Ascertainment of Exercise Status** (years) | 14.1 ± 10.4  [0.1, 61.3] | 8.4 ± 6.3  [0.7, 28.4] | 16.2 ± 7.7  [6.2, 34.0] | 8.3 ± 3.3  [2.8, 13.9] | <0.001 |
| **Wheezing Reported with Exercise** (% yes) | 23.9  (n = 909) | 20.8 | 12.5 | 21.4 | 0.59 |
| **Baseline FEV_1_ % Predicted†** | 84.2 ± 22.8  [19.7, 145.8]  (n = 707) | 89.5 ± 14.3  [48.4, 115.0]  (n = 26) | 81.4 ± 21.4  [35.1, 126.1]  (n = 24) | 101.9 ± 13.4  [78.8, 123.5]  (n = 9) | 0.06 |
| **Change in FEV_1_ % Predicted/Year††** | -1.33 ± 4.65  [-26.4, 24.3]  (n = 557) | 3.20  (n = 1) | (n = 0) | (n = 0) | 0.33 |
| **Baseline BMI Z-score†** | -0.16 ± 0.99  [-4.72, 3.12]  (n = 840) | 0.01 ± 0.98  [-2.72, 1.62]  (n = 33) | -0.03 ± 1.11  [-4.06, 1.99]  (n = 24) | 0.13 ± 1.01  [-1.39, 1.42]  (n = 7) | 0.59 |
| **Change in BMI Z-score/Year††** | -0.01 ± 0.20  [-1.02, 0.83]  (n = 590) | 0.18  (n = 1) | (n = 0) | (n = 0) | 0.37 |

**†**All FEV_1_ % predicted values were generated using U.S. CFF guidelines. All BMI Z-scores were generated using CDC percentiles. Baseline function represents the mean of all measurements obtained within 1 year before and after the age of exercise status ascertainment.

**††**Change in function represents the predicted change using linear regression and all measurements obtained 5 years after the age of exercise ascertainment for each subject. Only subjects with a minimum of 4 measurements and a minimum of 2 years of data were included.
